# Junior doctors’ early career choices do not predict career destination in neurology: 40 years of surveys of UK medical graduates

**DOI:** 10.1186/s12909-019-1650-7

**Published:** 2019-07-10

**Authors:** Atena Barat, Michael J. Goldacre, Trevor W. Lambert

**Affiliations:** UK Medical Careers Research Group, Unit of Health-Care Epidemiology, Nuffield Department of Population Health, University of Oxford, Old Road Campus, Oxford, OX3 7LF UK

**Keywords:** Neurology, Physicians, Junior, Career choice, Workforce, Medical, Medical education

## Abstract

**Background:**

The rapidly rising rates of brain diseases due to the growing ageing population and the explosion in treatment options for many neurological conditions increase the demand for neurologists. We report trends in doctors’ career choices for neurology; investigate factors driving their choices; and compare doctors’ original choices with their specialty destinations.

**Methods:**

A multi-cohort, multi-purpose nation-wide study using both online and postal questionnaires collected data on career choice, influencing factors, and career destinations. UK-trained doctors completed questionnaires at one, three, five, and ten years after qualification. They were classified into three groups: graduates of 1974–1983, graduates of 1993–2002, and graduates of 2005–2015.

**Results:**

Neurology was more popular among graduates of 2005–2015 than earlier graduates; however, its attraction for graduates of 2005–2015 doctors reduced over time from graduation. A higher percentage of men than women doctors chose neurology as their first career choice. For instance, among graduates of 2005–2015, 2.2% of men and 1.1% of women preferred neurology as first choice in year 1. The most influential factor on career choice was “enthusiasm for and commitment to the specialty” in all cohorts and all years after graduation. Only 39% who chose neurology in year 1 progressed to become neurologists later. Conversely, only 28% of practicing neurologists in our study had decided to become neurologists in their first year after qualification. By year 3 this figure had risen to 65%, and by year 5 to 76%.

**Conclusions:**

Career decision-making among UK medical graduates is complicated. Early choices for neurology were not highly predictive of career destinations. Some influential factors in this process were identified. Improving mentoring programmes to support medical graduates, provide career counselling, develop professionalism, and increase their interest in neurology were suggested.

**Electronic supplementary material:**

The online version of this article (10.1186/s12909-019-1650-7) contains supplementary material, which is available to authorized users.

## Background

As neurology is a medical specialty involving the diagnosis and treatment of patients with disorders of the nervous system [1], the demand for neurologists is growing faster than supply as a result of demographic changes which have increased the number of older individuals with degenerative neurological conditions; of accelerated progress in science, including new discoveries in functional brain anatomy; the invention of advanced techniques for making a precise diagnosis; and the advent of subspecialisations within neurology [2].

According to a World Health Organisation report, in 2017 Europe had the highest median number of neurologists per 100,000 population (9.0) [3]. In 2014, the UK had 1.2 consultant neurologists per 100,000 population [4]. In comparison, it was estimated that 16,366 neurologists were practising in the US in 2012, with a neurologist to population ratio of 5.2 per 100,000 [5].

Previous research showed several positive factors which can increase student recruitment into neurology, such as an interest in helping people [6], the doctors’ parents’ education level [7], effective mentorship [7–9], prior specialty experience [9, 10], early exposure to neurosciences [7] and the therapeutic bond between patient and doctor as a result of the chronic nature of neurological diseases [11]. In contrast, medical graduates’ interest in neurology can be deterred by financial considerations (e.g. personal debt and annual income) and ‘neurophobia’ [7]. Neurophobia is ascribed to deficiencies in medical education and is characterised by a fear of neuroscience and neurology due to the greater perceived difficulty of neuroscience and of neurological differential diagnosis compared to other specialties [10].

Understanding the factors which influence UK medical graduates’ decisions to choose neurology as their future career, and changes in their decisions, has a pivotal role in education and professional policies. The NHS, government agencies, specialist societies and medical Royal Colleges need to know about intending neurologists’ career choices and to make decisions about how to attract, select and retain these doctors, but at present such decisions lack an evidence base. The aims of the present paper were to report trends in medical graduates’ aspirations for a career in neurology; to identify factors influencing their choices for neurology; and to identify changes in their career pathway from their early choices to their eventual specialty destination. Such findings may help inform workforce planners to promote the recruitment, training and supply of the medical workforce in neurology.

It is worthwhile to note that this paper is one of a series of papers on individual specialties including anaesthesia [12], surgery [13], and cardiology [14] which are generated from our cohorts.

## Methods

### Aim, design, setting and participants

National longitudinal surveys were conducted by the UK Medical Careers Research Group from 1974 to 2015, collecting data contemporaneously through postal or web questionnaires from the UK medical graduates of 1974, 1977, 1980, 1983, 1993, 1996, 1999, 2000, 2002, 2005, 2008, 2009, 2011, 2012, and 2015. As the eligibility criterion for inclusion in the surveys is being a registered doctor, subjects’ contact details were provided by the General Medical Council (GMC) with whom all doctors who wish to practise in the UK must be registered.

The surveys used a multi-purpose questionnaire at different time slots: 1, 3, and 5 years after primary medical qualification (graduation from medical school) and at longer intervals thereafter. This paper focuses on three concepts: early career preferences for eventual specialty, factors influencing career preferences, and career specialty destinations. Data on career preferences and influencing factors was analysed from surveys of doctors 1, 3, and 5 years after graduation, using, respectively, 15 cohorts (1974–2015), 12 cohorts (1974–2008 and 2012), and 10 cohorts (1974–1980 and 1993–2008). Data on career destinations was collected from the doctors 10 years after graduation, using 5 cohorts (1993–2002).

Participation was voluntary and required completing a self-administered questionnaire. To maximise the response rate, all non-respondents received up to four paper or email reminders over the ensuing months following the initial contact.

Further details of the research method are presented elsewhere [15].

### Research instrument

The content of the research instrument has been established in usage over many years. Broadly, the questions explored demographic information, career choices and plans, and employment history.

The questionnaire utilised structured questions, for example: “What is your choice of long-term career?” Respondents were invited to specify up to three choices of specialty for their future career in order of preference, identifying choices of equal preference (referred to as “tied choices”). In other words, a tied neurology first choice means that a participant’s first choice was to follow either neurology or another named specialty. Then, we asked whether their choice of specialty is “definite”, “probable” or “uncertain”. They were also asked to specify the importance of each of 13 listed factors in influencing their specialty choice: “not at all”, “a little” or “a great deal”.

### Statistical analyses

For simplicity, in most tables we combined data on choices into three cohort groups: graduates of 1974–1983, graduates of 1993–2002, and graduates of 2005–2015.

All data were processed and analysed using IBM SPSS Statistics for Windows, V22 [16] and Microsoft Excel (2010). We assessed the association between binary or unordered categorical variables by univariate analysis with χ^2^ tests. To identify linear trends over cohorts, the Mantel–Haenszel linear-by-linear χ^2^ test was applied. In this paper, 95% confidence intervals were quoted for the main results.

## Results

### Response rate

The response rates for cohorts included in this paper were 56.9% (40,412/71,026) in the first year after graduation, 62.3% (31,466/50,477) in year 3 and 55.4% (24,970/45,071) in year 5.

### First choice of neurology one, three and five years after graduation

Over all cohorts combined, in years 1, 3, and 5 respectively, 1.0, 0.9, and 0.9% of responders made neurology their first choice. In year 1 there was a substantial linear increase in choices for neurology across the cohort groupings: only 0.6% of the graduates of 1974–1983 chose neurology, compared with 0.9% of the graduates of 1993–2002 and 1.5% of the graduates of 2005–2015. In years 3 and 5 this trend was less evident, though graduates from the year 1993 onwards showed a higher level of choices for neurology than did those of 1974–83. Table 1 shows comparative figures and the results of statistical comparisons.

**Table 1:** Trends among UK medical graduates in early first choices for eventual careers in neurology

Year after graduation	Cohorts (years of graduation)				Statistical tests	
	1974–1983	1993–2002	2005–2015	All cohorts	Linear trend (χ^2^_1_, p)	Heterogeneity (χ^2^_2_, p)
Year 1	0.6%	0.9%	1.5%	1.0%	49.8, < 0.001	52.6, < 0.001
Year 3	0.7%	1.1%	0.9%	0.9%	2.9, 0.09	11.9, 0.003
Year 5	0.7%	1.0%	0.9%	0.9%	1.0, 0.32	2.8, 0.25

For doctors surveyed one, three and five years after graduation the table shows the percentages of doctors who specified neurology as their first choice of eventual specialty

**Fig. 1:**
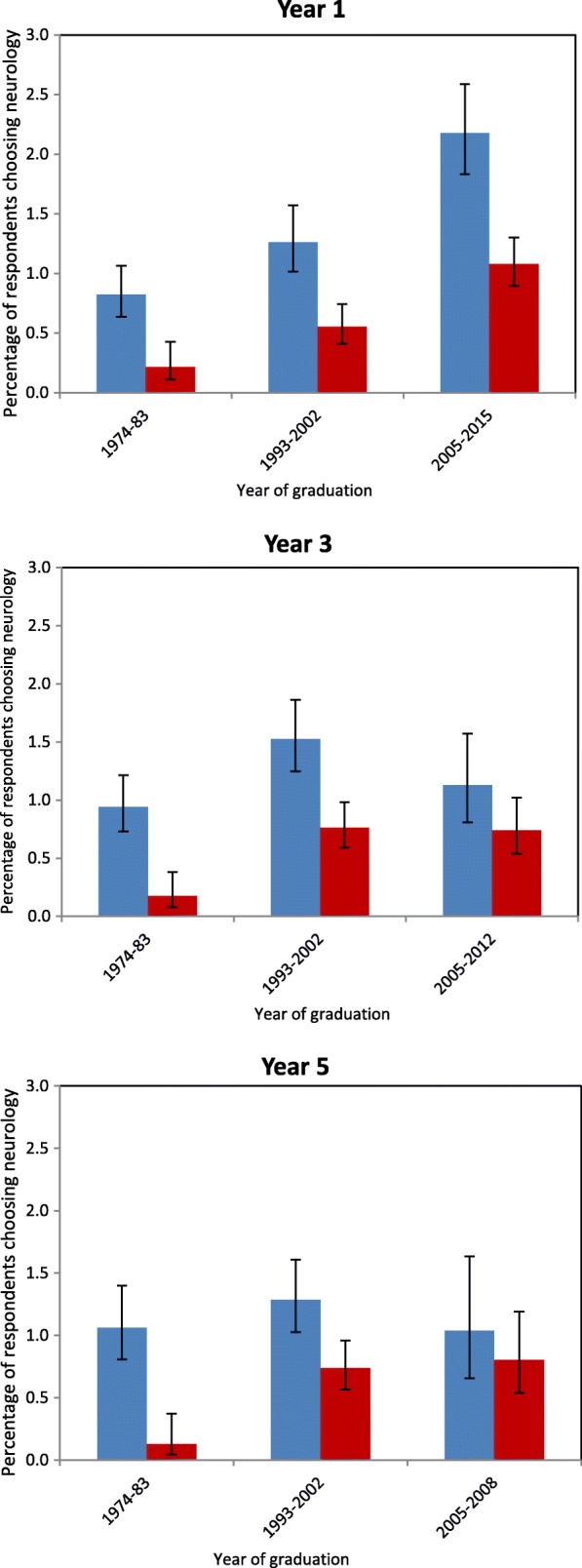
Choosing neurology as first choice of eventual career in year 1, 3, and 5 after graduation

There were large differences between the percentages of men and of women who chose neurology, in all the cohort groupings. For example, in year 1 among the graduates of 2005–2015, 1.1% (CI: 0.9 to 1.3%) of women chose neurology as their first choice compared with 2.2% (1.8 to 2.6%) of men. For years 3 and 5 respectively the corresponding figures for the most recent cohort group were: year 3 women 0.7% (0.5 to 1.0%), men 1.1% (0.8 to 1.6%); year 5 women 0.8% (0.5 to 1.2%), men 1.0% (0.7 to 1.6%). Figure 1 illustrates the level of first choices for neurology of men and women in each cohort grouping, at years 1, 3, and 5. Conventional 95% confidence intervals are shown on the figure as a guide to assessing the significance of the male-female differences.

**Fig. 2:**
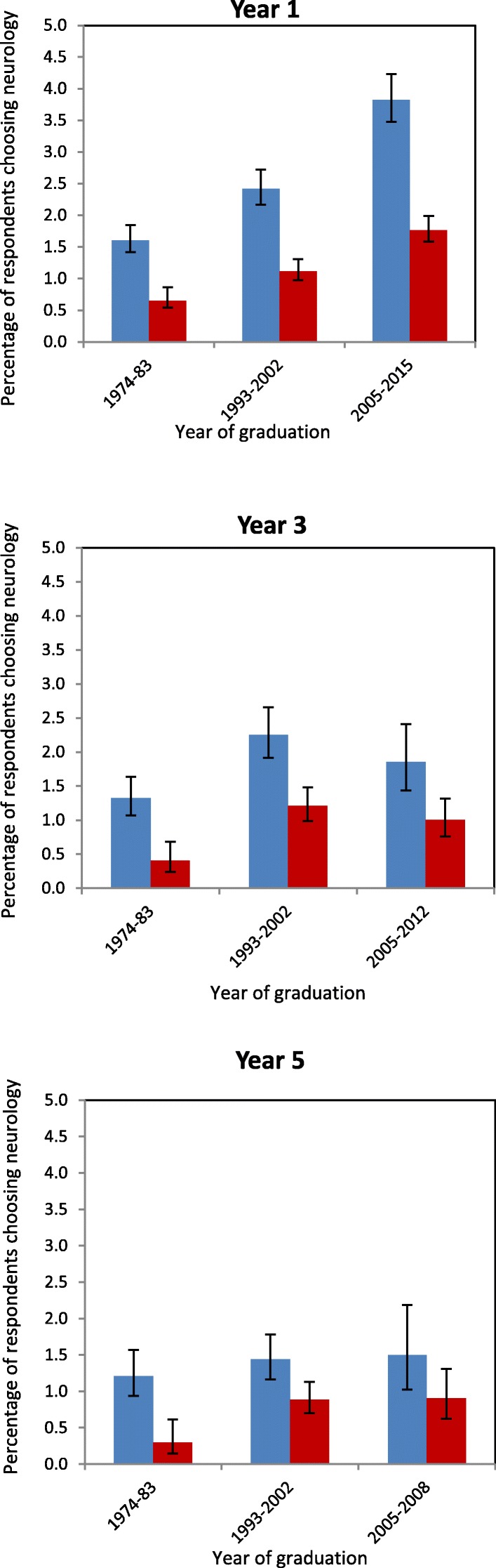
Choosing neurology as any choice of eventual career in year 1, 3, and 5 after graduation

Appendix: Figure 2 shows the corresponding results for men and women who made *any choice* for neurology – in other words not solely first choices, but also including those who made neurology their second or third choice.

For the most recently surveyed individual cohorts, we present Additional file 1: Table S1 (see Appendix) showing the percentages who chose neurology in each cohort survey. Year 1 first choices varied from 1.2% in the 2012 and 2015 cohorts to 2.0% in the 2009 cohort. For the cohorts surveyed, year 3 and year 5 choices also showed large variation from cohort to cohort.

Table 2 compares the level of certainty of career choice between doctors who chose neurology as their first choice with those who chose other hospital physician specialties. In Year 1, 19.7% of those who considered neurology as their first choice were definite about their decision. Their certainty rose to 32.9% and 67.4% in years 3 and 5, respectively. This trend was less pronounced among participants whose first choice was another hospital physician specialty: in year 1 their certainty of career choice was 12.7% (significantly less than those who chose neurology, *p* < .001), but it increased in years 3 and 5 (to 25.3% and then to 60.7%). In all years, there was no significant difference between male and female doctors in the level of their certainty about neurology as their first career choice (*p* > 0.01 using χ^2^ test).

**Table 2:** Certainty of career choice by specialty chosen one, three and five years after graduation: numbers (N) and percentages (%) of respondents

		Level of certainty about career choice			
Year after graduation	Specialty choice	Definite N(%)	Probable N(%)	Uncertain N(%)	Total N(%)
Year 1	Neurology	81 (19.7)	236 (57.4)	94 (22.9)	411 (100)
	Other hospital physician specialties	1191 (12.7)	4663 (49.9)	3498 (37.4)	9352 (100)
Year 3	Neurology	93 (32.9)	133 (47.0)	57 (20.1)	283 (100.0)
	Other hospital physician specialties	1220 (25.3)	2363 (49.1)	1233 (25.6)	4816 (100.0)
Year 5	Neurology	149 (67.4)	59 (26.7)	13 (5.9)	221 (100.0)
	Other hospital physician specialties	2079 (60.7)	1074 (31.4)	270 (7.9)	3423 (100.0)

χ^2^_2_ test results, comparing those who chose neurology with those who chose other hospital physician specialties: Year 1 χ^2^_2_ = 41.8, p < .001; Year 3 χ^2^_2_ = 9.3, *p* = .010; Year 5 χ^2^_2_ = 4.1, *p* = .132

In this paper “other hospital physician specialties” comprises general medicine, cardiology, dermatology, endocrinology, geriatrics, nephrology, chest medicine, rheumatology/rehabilitation, academic medicine, genito-urinary medicine (or venereology), gastroenterology, vascular medicine, tropical medicine, clinical pharmacology, infectious diseases, occupational medicine (or industrial medicine)

### Influencing factors on career choice

Thirteen possible influencing factors on doctors’ career choice were identified by reviewing available literature. Table 3 reflects the responses of participants regarding factors with a great deal of influence on their career choice.

**Table 3:** UK doctors who specified each factor as influencing their choice of career “a great deal”

Factor influencing career choice	Year 1		Year 3		Year 5	
	Neurology N (%)	Other hospital physician specialties N (%)	Neurology N (%)	Other hospital physician specialties N (%)	Neurology N (%)	Other hospital physician specialties N (%)
Domestic situation	62 (19.5*)	1738 (24.9)	36 (17.6*)	801 (23.6)	53 (24.2)	921 (28.0)
Hours/working conditions	79 (27.9)	1925 (31.9)	68 (48.2**)	806 (36.3)	69 (41.6)	1027 (39.9)
Future financial prospects	14 (6.0)	455 (8.4)	8 (5.7)	192 (8.6)	9 (5.4)	208 (8.0)
Financial circumstances whilst training	4 (11.8**)	30 (3.1)	3 (3.0)	85 (5.3)	5 (6.7)	69 (6.0)
Career/promotion prospects	48 (17.9)	1282 (20.1)	48 (28.2)	833 (28.8)	53 (24.0)	856 (25.9)
Self-appraisal	132 (49.3)	2927 (45.8)	119 (58.3)	1855 (54.8)	121 (55.0)	1885 (57.0)
Advice from others	25 (9.3**)	997 (15.6)	29 (17.2)	584 (20.2)	38 (17.2)	546 (16.5)
Student experience of subject	161 (50.6***)	2590 (37.0)	71 (39.0***)	814 (25.6)	76 (37.4***)	701 (22.5)
Particular teacher/department	90 (33.6**)	1679 (26.3)	62 (42.2)	984 (36.1)	66 (36.5)	1028 (38.2)
Inclinations before medical school	44 (16.4***)	501 (7.8)	39 (21.5***)	220 (6.9)	28 (13.9***)	197 (6.3)
Experience of jobs so far	77 (33.0***)	2794 (51.3)	113 (55.1)	1973 (58.5)	140 (63.6)	2163 (65.4)
Enthusiasm/commitment	183 (64.9*)	3473 (57.9)	100 (71.9)	1540 (69.6)	136 (81.4)	2071 (80.1)
Other reasons	22 (16.4**)	249 (9.1)	14 (14.3)	263 (18.0)	24 (26.1)	268 (23.9)

Key: * indicates *p* < .05; ** indicates *p* < .01; *** indicates *p* < .001, comparing neurology with other hospital physician specialties, within each year, for each factor

“Enthusiasm/commitment: what I really want to do” was considered as the most influential factor on career choice among all participants (i.e. those choosing neurology or other hospital physician specialties as their first choice of eventual career) although the intending neurologists did not score substantially higher than others on “enthusiasm/commitment” in any of 1, 3, and 5 years after graduation.

The second influential factor for doctors who chose neurology was “student experience of subject” (50.6%) in year 1, “self–appraisal of own skills/aptitudes” (58.3%) in year 3 and “experience of jobs so far” (63.6%) in year 5 after graduation. For respondents who chose other hospital physician specialties “experience of jobs so far” was their second most influential factor, with increasing importance, in year 1 (51.3%), 3 (58.5%) and 5 (65.4%) after graduation.

“Student experience of subject” and “inclinations before medical school” were the only factors to be consistently rated as significantly (*p* < .001) more important to intending neurologists than other hospital physician specialists, in all three survey years.

We compared the scoring of importance of the factors by men and women who chose neurology (results not shown). Female doctors who considered neurology as a career were more concerned than their male peers about “wanting a career that fits my domestic situation” at year 1 after graduation (*p* = 0.006, χ^2^ = 7.6). In comparison, at year 3, “career and promotion prospects” were more important for men who had specified neurology as a first choice than for women (*p* = 0.001, χ^2^ = 11.3). Further analysis of all factors did not show any other significant differences between male and female intending neurologists.

### Specialty destination

Ten years after graduation, the doctors from the cohorts of 1993 to 2002 were categorized according to whether they were now practicing neurologists (Tables 4 and 5 ).

**Table 4:** Career destinations at 10 years of doctors who expressed a career preference for neurology in years 1, 3, or 5

	Year 10 destinations			
	Neurology N (%)	Other hospital physician specialties N (%)	Others N (%)	All destinations N (%)
Year 1 1st choices				
Men choosing neurology	29 (47.5)	16 (26.2)	16 (26.2)	61 (100.0)
Women choosing neurology	8 (24.2)	13 (39.4)	12 (36.4)	33 (100.0)
Total choosing neurology	37 (39.4)	29 (30.9)	28 (29.8)	94 (100.0)
Year 3 1st choices				
Men choosing neurology	54 (70.1)	10 (13.0)	13 (16.9)	77 (100.0)
Women choosing neurology	22 (50.0)	12 (27.3)	10 (22.7)	44 (100.0)
Total choosing neurology	76 (62.8)	22 (18.2)	23 (19.0)	121 (100.0)
Year 5 1st choices				
Men choosing neurology	61 (95.3)	0 (0.0)	3 (4.7)	64 (100.0)
Women choosing neurology	32 (74.4)	5 (11.6)	6 (14.0)	43 (100.0)
Total choosing neurology	93 (86.9)	5 (4.7)	9 (8.4)	107 (100.0)

*p*-values, comparing the percentages of men and women who chose neurology in each year who later worked as neurologists: year 1 *p* = .047; year 3 *p* = .045; year 5 *p* = .004

**Table 5:** Original career choices in years 1, 3, and 5 of doctors practising as neurologists in year 10

Career choices	Male N (%)	Female N (%)	Total N (%)
Year 1			
Neurology untied 1st choice	23 (32.3)	7 (18.9)	30 (27.7)
Neurology tied 1st choice	6 (8.4)	1 (2.7)	7 (6.4)
Neurology 2nd or 3rd choice	7 (9.8)	3 (8.1)	10 (9.2)
Any choice for other hospital physician specialties	26 (36.6)	14 (37.8)	40 (37.03)
Other choices	9 (12.6)	12 (32.4)	21 (19.4)
Total	71 (100.0)	37 (100.0)	108 (100.0)
Year 3			
Neurology untied 1st choice	47 (70.1)	20 (55.5)	67 (65.04)
Neurology tied 1st choice	7 (10.4)	2 (5.5)	9 (8.7)
Neurology 2nd or 3rd choice	2 (2.9)	2 (5.5)	4 (3.8)
Any choice for other hospital physician specialties	7 (10.4)	6 (16.6)	13 (12.6)
Other choices	4 (5.9)	6 (16.6)	10 (9.7)
Total	67 (100.0)	36 (100.0)	103 (100.0)
Year 5			
Neurology untied 1st choice	56 (75.6)	32 (76.1)	88 (75.8)
Neurology tied 1st choice	5 (6.7)	0 (0.0)	5 (4.3)
Neurology 2nd or 3rd choice	7 (9.4)	3 (7.1)	10 (8.6)
Any choice for other hospital physician specialties	5 (6.7)	3 (7.1)	8 (6.8)
Other choices	1 (1.3)	4 (9.5)	5 (4.3)
Total	74 (100.0)	42 (100.0)	116 (100.0)

Data are for neurologists at ten years after medical school graduation (1993, 1996, 1999, 2000, 2002 cohorts)

Looking ‘forward’ from early choices to career destinations (Table 4), 39.4% of those who chose neurology as a first choice in year 1 were working as neurologists in year 10, compared with 62.8% for year 3 choices and 86.9% for year 5 choices. Gender differences were significant: in each year proportionately fewer of the women than the men who chose neurology in year 1 worked in the specialty later, and even for those who at year 5 intended to be neurologists a quarter of women were not working in it five years later (Table 4).

Looking ‘backwards’ from career destinations ten years after graduation to earlier choices (Table 5), of those working in neurology at year 10, more than a quarter (27.7%) had specified it as their sole first choice in year 1; but this percentage increased substantially at year 3 and reached over three-quarters (75.8%) when year 5 choices were considered. The same trend was observed for males and females: χ^2^_1_ tests, comparing the percentage of men and women neurologists who had made it a first choice in each of years 1, 3, and 5, gave non-significant results (*p* = .07, .056, .570 respectively). The percentage of practising neurologists who had chosen other hospital physician specialties in year 1 was 37.0% compared with 6.8% when year 5 choices were considered.

## Discussion

### The context of specialist training in neurology in the UK and the US

Presently, specialty training in neurology in the UK takes five years, one of which may be relevant research. Entry into neurology training is possible following successful completion of a two-year postgraduate foundation programme, followed by a three-year core training programme. There are two core training programmes in the UK which are relevant to neurology training: Core Medical Training, and Acute Care Common Stem - Acute Medicine. A fully qualified neurologist in the UK will therefore have completed an undergraduate medical degree of typically three to five years in duration, a two year postgraduate foundation, a three year core training, and a five-year specialist neurology training, a total of some 13–15 years. In the context of our study, at year 1 we surveyed doctors in their first foundation year, in year 3 in their first year of core training, and in year 5 at the end of core training, as they make a commitment to a particular specialty in which to complete their training.

For comparison, the training system in the US is broadly similar, though there are terminological differences. Typically, a four year medical degree would be followed by a one to three-year internship in internal medicine then followed by 5 to 7 years of specialist training in neurology.

### Main findings of this study

The popularity of neurology was higher one year after graduation among recent medical graduates (2005–2015) than among their predecessors; nevertheless, unlike earlier cohorts, as more time passes from their graduation, the level of attraction towards neurology fell among these young graduates. Among those who chose neurology in year 1, certainty about their choice was higher than among those who chose other hospital physician specialties, but this difference reduced in years 3 and 5. High competition ratios for neurology residency positions (four candidates per specialty training post has been quoted in 2016) [17] or more appealing conditions of work in other specialties might underpin the above trends.

This paper found neurology to be a male-dominated specialty, in all cohorts and years from graduation, although there was some evidence from the most recent cohorts surveyed at year 5 that the gender difference may be reducing. Having chosen neurology, women were less likely than men to convert their choice into a career in neurology; and, even as late as five years after graduation, women specifying neurology as their first choice were less likely than men to be working in the field five years later. Gender imbalance in medical specialties and particularly in neurology was frequently reported in other national and international studies [10, 18, 19]. One possible explanation for this can be the perceived difficulty to maintain work-life balance among neurologists [10] and stereotypical assumptions about what women should do [20]. As the family life of female doctors is more affected than that of male doctors by their work [20, 21], the reported gender differences are to some degree understandable.

The correspondence between early career choices and eventual specialty destinations was quite strong for neurology, especially for men, but not as strong as other specialties. For example, 39% of those who chose neurology in year 1 were neurologists in year 10. As comparisons in other specialties, 82% of doctors who chose general practice in year 1 were general practitioners in year 10; 75% who chose psychiatry in year 1 were psychiatrists in year 10. Conversely, 30% of practising neurologists had chosen neurology as their preferred career in year 1; 50.0% of practising GPs in year 10 had chosen general practice as their preferred career in year 1; and 52% of practising psychiatrists had specified psychiatry as their preferred career choice in year 1 [22]. Thus, all these specialties were boosted by ‘late converters’, doctors who did not choose the specialties in the early years but who eventually practised in them.

Decisions about neurology as an eventual career were still uncertain in a quarter of cases five years after graduation. This problem was similarly reported in other medical specialties such as cardiology [14] and results in late decisions to commit a career. It may be appropriate for decisions to commit to individual specialties within physician practice to be deferred; but this is a matter for discussion and decision within the profession. Consideration should be given to whether late decision-making might be attributed to the lack of knowledge about the reality of working in a specialty (in this case neurology) among medical graduates and insufficient support for them to choose a career that best matches their aptitude and willingness [7].

In this study, “enthusiasm/commitment”, “student experience of subject”, “experience of jobs so far”, and “self-appraisal” were identified as the most influential factors which can inspire medical graduates to neurology. It is notable that the first two factors were also emphasised by our participants who intended to pursue other hospital physician specialties. Since core professional training for neurology and other hospital physician specialties is the same [23], it is rational that their applicants have common motivations and inspirations.

Advantages of close agreement between early choices and later destinations in neurology are that early specialist training programmes can be organised in the expectation that early choosers will hold their enthusiasm and fascination for the specialty in the longer term; and that, if past performance is a guide to future opportunities, that they will be successful in getting jobs in the specialty. A case against early specialist training programmes confined to neurology is that they may reduce future career options, if needed, in other branches of specialist physician practice. These are issues for consideration by trainers, trainees and educational planners within neurology and general physician practice.

### Implications

This paper offers insights into neurology career choices and progression through examining its recruitment trends, gender differences in the specialty, influencing factors on career decision, and the matching of original choices and later career destinations among practising neurologists. As the data reveal, neurology holds greater appeal for recent medical graduates than their predecessors. It is important to maintain this trend and increase its rate over the coming years to meet the current and future needs of neurological patients. In recent years, the number of female doctors choosing neurology as their future career has significantly increased but the gender gap still persists. Adopting some innovative policies may tackle this gender imbalance in the specialty and help female doctors reach their full potential. Such policies may include encouraging family friendly benefits, offering paid parental leave, and flexible working hours [24].

Study findings about influencing factors are in line with previous studies that highlighted the importance of interest and fascination [11], and prior experience factors [7, 10] for intending neurologists. The inherent interest and fascination factor, usually regarded as “good fit”, is an intangible element [11] and can be shaped through the effect of other elusive factors. None of the previous work has examined the impact of this factor on medical students’ thinking regarding their career choice. Therefore, further studies are required to provide a more sophisticated and in-depth exploration of its components and compare them between neurology and other specialties.

Addressing late career decisions by improving mentorship programmes may be one strategy to accelerate the career decision process and subsequently promote satisfaction and retention for intending neurologists. The programme should embed early socialization strategies such as job-shadowing to provide realistic and contemporary portrayals of neurology practice and enhance the applicants’ understanding of their future practice roles and care environment [25]. CORTEX (Comprehensive Opportunities for Research and Teaching Experience) developed by Zuzuárregui and Hohler in the US is an example of a successful longitudinal mentorship programme that constitutes a consistent interaction between students and mentors to increase the exposure of students to neurology through teaching and research [26]. Further research is suggested to design a practice development plan for medical students which focuses on individuals’ abilities while considering contextual variables [27, 28].

### Limitations and strengths

Despite the high overall response rate of the study, non-response bias is possible.

This is a unique, large-scale, national, and multi-cohort study in the UK that captured the views and aspirations of medical graduates over many years (1974–2015). It has produced a big database of information which can be applied for generating evidence relevant to policies and planning in other individual specialties regarding medical training and doctors’ recruitment in the UK health care system.

## Conclusions

This paper adds to the available information on neurology careers in the UK. The study findings should be helpful for medical graduates considering neurology for their eventual career as well as education providers, decision makers, commissioners of services, workforce planners and other stakeholders. The latter can consider the implications of this paper and use them in preparing and supporting medical graduates for their future careers. These may help in planning service provision and the supply of an adequate professional workforce for people affected by neurological conditions in the UK.

### Additional file

Additional file 1: **Table S1.** Choices for neurology in individual recent cohorts: percentages and numbers of responders. (DOCX 16 kb)

## Data Availability

The datasets generated and/or analysed during the current study are not publicly available due to reasons of practicality. It may be possible for the authors to make tabulated data, produced in the course of this work but not included in the paper, available to interested readers on request.

## Abbreviations

CI: Confidence interval
CORTEX: Comprehensive Opportunities for Research and Teaching Experience
GMC: General Medical Council
GP: General practitioner (family doctor)
NHS: National Health Service of the United Kingdom
UK: United Kingdom
US: United States of America
