# Supplementary material for: Junior doctors’ early career choices do not predict career destination in neurology: 40 years of surveys of UK medical graduates
Source: BMC Med Educ. 2019 Jul 10;19:257. doi: 10.1186/s12909-019-1650-7 (PMC6621943; doi:10.1186/s12909-019-1650-7)
Supplement: Supplementary file 1 — Table S1. Choices for neurology in individual recent cohorts: percentages and numbers of responders. (DOCX 16 kb) [file 12909_2019_1650_MOESM1_ESM.docx]

Additional file 1: Table S1 Choices for neurology in individual recent cohorts: percentages and numbers of responders

| Graduation year | Year after graduation | | |
| --- | --- | --- | --- |
|  | **Year 1**  **% (N)** | **Year 3**  **% (N)** | **Year 5**  **% (N)** |
| First choice | | | |
| 2005 | 1.3 (40/3128) | 0.8 (21/2710) | 0.6 (15/2362) |
| 2008 | 1.6 (54/3302) | 1.1 (36/3228) | 1.1 (27/2369) |
| 2009 | 2.0 (57/2917) | no survey | no survey |
| 2011 | 1.8 (18/1001) | no survey | no survey |
| 2012 | 1.2 (29/2398) | 0.7 (14/2063) | no survey |
| 2015 | 1.2 (36/3040) | no survey | no survey |
| Any choice | | | |
| 2005 | 2.1 (66/3128) | 1.2 (33/2710) | 0.7 (17/2362) |
| 2008 | 3.0 (100/3302) | 1.6 (53/3228) | 1.5 (36/2369) |
| 2009 | 2.8 (81/2917) | no survey | no survey |
| 2011 | 3.1 (31/1001) | no survey | no survey |
| 2012 | 2.5 (59/2398) | 1.0 (20/2063) | no survey |
| 2015 | 2.0 (61/3040) | no survey | no survey |

**Key:** “Any choice” means neurology was selected as either a first, second or third choice of eventual career
